# Early Molecular Events during Onset of Diapause in Silkworm Eggs Revealed by Transcriptome Analysis

**DOI:** 10.3390/ijms21176180

**Published:** 2020-08-27

**Authors:** Jing Gong, Xi Zheng, Shan Zhao, Lingzhen Yang, Zhao Xue, Zhengjie Fan, Miao Tang

**Affiliations:** State Key Laboratory of Silkworm Genome Biology, College of Sericulture, Textile and Biomass Sciences, Southwest University, Chongqing 400715, China; pluto_zx@126.com (X.Z.); zs104986@email.swu.edu.cn (S.Z.); y18223115132@email.swu.edu.cn (L.Y.); xuexinan@email.swu.edu.cn (Z.X.); fzj974377312@email.swu.edu.cn (Z.F.); tm46720@163.com (M.T.)

**Keywords:** *Bombyx mori*, diapause, oxidative phosphorylation pathway, insulin/FoxO signaling pathway, RNA-sequencing

## Abstract

Diapause is a form of dormancy, and *Bombyx mori* silkworm embryos are ideal models for studying diapause in insects. However, molecular events in eggs during the onset of diapause remain unclear. In this study, transcriptome analyses were performed on silkworm diapause eggs via RNA sequencing at 20 and 48 h after oviposition. A total of 6402 differentially expressed genes (DEGs) were detected in diapause eggs at 48 h versus that at 20 h after oviposition. Gene ontology enrichment analysis showed that DEGs in diapause eggs at 48 h versus that at 20 h after oviposition were involved in ribosome-related metabolism and hydrogen transport. Kyoto Encyclopedia of Genes and Genomes analysis revealed several significantly enriched biological pathways, namely the oxidative phosphorylation, Forkhead box protein O3 (FoxO) signaling, ribosome, endoplasmic reticular protein processing, and autophagy pathways. Fifteen DEGs from the FoxO signaling pathway were selected, and their expression profiles were consistent with the transcriptome results from real-time quantitative reverse transcription polymerase chain reaction. Our results can improve understanding of the diapause mechanism in silkworm eggs and identified key pathways for future studies.

## 1. Introduction

The external environment is not always suitable for the growth of organisms, especially the case in ectotherms. Dormancy is a strategy that enables bacteria, plants, and animals to survive and reproduce by minimizing metabolic activity to conserve energy. Diapause is a form of dormancy involving predictability [1], metabolic inhibition, developmental arrest, and enhanced stress tolerance [2]. It also involves various “programmed” physiological and molecular processes. Organisms can switch their developmental state to a diapause state to survive and/or reproduce under seasonal variations. Numerous animals, including nematodes, insects, fish, and mammals [2,3,4,5], exhibit this phenomenon.

*Bombyx mori* (silkworm) is an economically important insect used in silk production. Silkworm rearing has a history of more than 5000 years in China, and sericulture is still practiced in China, India, and Italy, among others. Silkworm genetics and genomics have been extensively researched, and these insects provide insights into embryonic diapause. Diapause hormone (DH), a neuropeptide hormone secreted by the maternal sub-esophageal ganglion, induces eggs to enter diapause [6]. *Bombyx* transient receptor potential A1 (*BmTrpA1*) is the central regulator that determines transgenerational DH release [7]. Controlled by DH, glycogen accumulates in developing ovaries, affecting the induction of diapause in progeny [8]. After oviposition, diapause-destined eggs gradually enter diapause for 10 days, with a series of dramatic changes occurring during the onset of diapause. For example, the color of diapause eggs changes from pale yellow to pale red within 48 h of oviposition [9], and the embryo is in the telson formation stage of embryogenesis, just before diapause phase I [10]. Oxygen consumption of diapause eggs peaks at 24 h after oviposition and rapidly decreases after 48 h [11]. Glycogen in diapause eggs is rapidly converted to sorbitol and glycerol during this phase [12].

Our previous studies on silkworm eggs via RNA sequencing showed that the gene expression patterns of hyperoxia- and HCl-treated diapause eggs were similar, which differed from non-treated diapause eggs [13]. Further physiological research has shown that reactive oxygen species (ROS) levels increase markedly in diapause eggs during this phase, but remain stable in non-diapause eggs, indicating that ROS may play a key role in promoting the onset of diapause [14]. However, the molecular events in eggs during the onset of diapause remain unclear. To explore the embryonic development of silkworm diapause eggs, we performed transcriptome analyses on diapause eggs during onset of diapause via RNA sequencing. The differentially expressed genes (DEGs) of diapause eggs at 48 h versus that at 20 h after oviposition were analyzed, and important pathways, such as the oxidative phosphorylation pathway, related to diapause were revealed. These results may improve understanding of the molecular mechanism of the onset of diapause in silkworm eggs and those of other insects.

## 2. Results

### 2.1. Embryo Development 24–48 h after Oviposition

**Figure 1:**
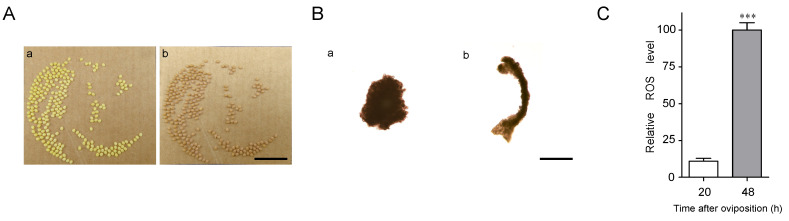
Differences in silkworm diapause eggs at 20 and 48 h after oviposition. (**A**) Egg color at (a) 20 h and (b) 48 h. Bars represent 10 mm. (**B**) Embryos at (a) 20 h and (b) 48 h. Bars represent 400 µm. (**C**) Difference in reactive oxygen species (ROS) levels. For ROS detection, approximately 60 eggs were sampled at a time. Relative ROS levels indicated fluorescence intensity/protein concentration (mg/mL), with 100 as the highest value. Each point represents mean ± SD of three independent replicates. ***, *p* < 0.001.

Diapause-destined eggs gradually entered diapause 24–48 h after oviposition. The color of these eggs changed from pale yellow at 20 h after oviposition to pale red at 48 h (Figure 1A). To analyze embryonic development during this period, silkworm eggs were dissected. The embryos were at the cephalic lobe formation stage and showed a pyriform-shape at 20 h after oviposition (Figure 1B). They were significantly elongated, and their anterior and posterior segments became distinguishable, entering the telson formation stage at 48 h after oviposition (Figure 1B). To compare the physiological conditions in the diapause eggs, ROS levels were measured between 20 and 48 h after oviposition. ROS levels were significantly higher at 48 h than at 20 h (Figure 1C).

### 2.2. RNA Sequencing

To explore the diapause mechanism, we performed RNA sequencing and obtained 56,668,126, 55,326,462, and 54,874,326 clean reads from diapause eggs at 20 h after oviposition and 60,410,096, 53,811,608, and 42,353,288 clean reads from those at 48 h after oviposition (Table 1). The read counts were converted to expected number of fragments per kilobase of transcript sequence per million base pairs sequenced (FPKM) (Table S1) and showed that the gene expression levels of the eggs were similarly distributed at 20 and 48 h after oviposition (Figure S1). The proportion of total reads that mapped to the reference genome ranged from 96.02% to 97.07% (Table 1), and the correlation values were significantly higher between the duplicated samples than among the groups (Figure S2).

### 2.3. Detection of DEGs

**Figure 2:**
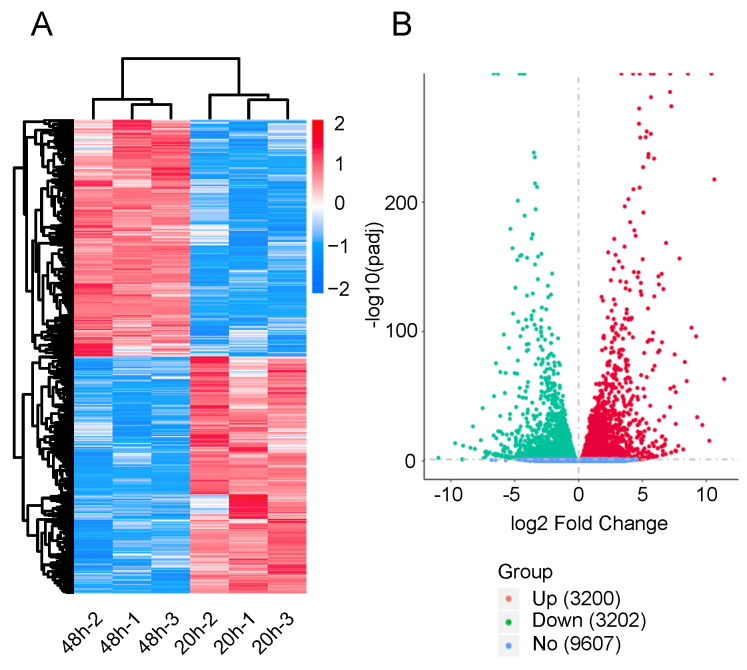
Hierarchical cluster analysis and volcano plot of differentially expressed genes (DEGs) in silkworm diapause eggs at 48 h versus that at 20 h after oviposition. (**A**) Hierarchical clustering of DEGs. Blue indicates low expression, white indicates moderate expression, and red indicates high expression. (**B**) Volcano plot of DEGs. Red, green, and blue dots represent upregulated, downregulated, and unchanged genes, respectively. The number in brackets is the number of DEGs.

To determine the gene expression patterns of diapause eggs at 20 and 48 h after oviposition, hierarchical clustering was performed based on the FPKMs. The gene expression patterns of diapause eggs at 20 h differed from those at 48 h, but were similar in the duplicated samples (Figure 2A). A total of 6402 DEGs were detected in diapause eggs at 48 h compared with 20 h, among which 3200 DEGs had upregulated expression and 3202 had downregulated expression. Furthermore, 9607 genes showed no differential expression (Figure 2B, Table S2).

### 2.4. Gene Ontology Analysis of DEGs

**Figure 3:**
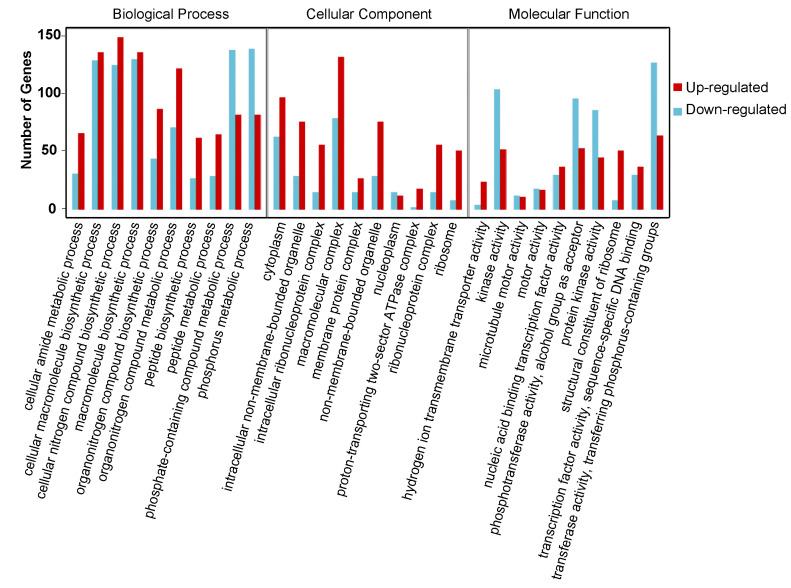
Gene ontology (GO) categories of common differentially expressed genes in silkworm diapause eggs at 48 h versus that at 20 h after oviposition. *Y*-axis represents number and percentage of unigenes mapped to indicated GO term, and *x*-axis represents each GO term. Red and blue bars represent upregulated and downregulated genes, respectively. GO terms with an adjusted *p*-value of <0.05 were considered significantly enriched.

To clarify the function of DEGs involved in the onset of diapause, all the DEGs were mapped to the whole transcriptome background and given gene ontology (GO) orthology annotations. GO analysis showed that 5597 genes (87.43% of all DEGs) could be subcategorized into 735 hierarchically structured GO classes in diapause eggs at 48 h versus that at 20 h after oviposition (Figure 3, Table S3), and 54 significantly enriched pathways were analyzed. In the biological process, 41 GO pathways were identified, including eight related to phosphate metabolism and transport, four to protein metabolism, and two to hydrogen transport. In the cellular component domain, three related to ribosome pathways, two to non-membrane-bounded organelle pathways, and one to protein modification were identified. In the molecular function domain, one pathway related to ribosome structural constituents and six other pathways were identified (Table S3).

### 2.5. Kyoto Encyclopedia of Genes and Genomes Enrichment Analysis of DEGs

**Figure 4:**
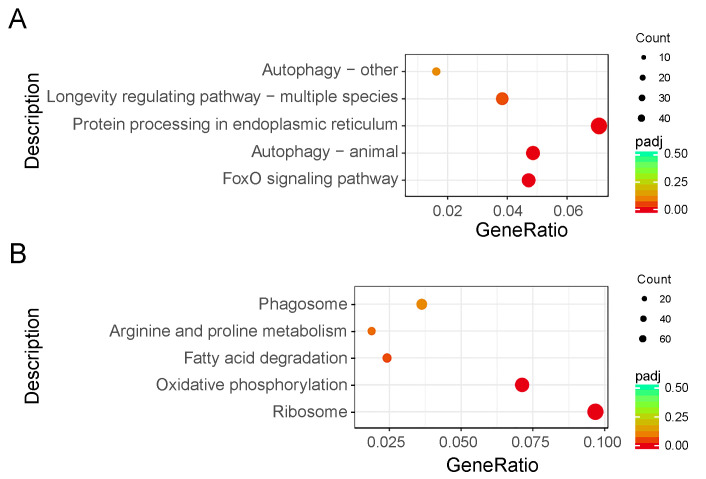
Kyoto Encyclopedia of Genes and Genomes enrichment scatter plot of differentially expressed genes (DEGs) in silkworm diapause eggs at 48 h versus that at 20 h after oviposition. *Y*-axis represents name of pathway, and *x*-axis represents rich factor. Dot size represents number of different genes, and color indicates adjusted *p*-value. (**A**) Downregulated DEG enrichment analysis results. (**B**) Upregulated DEG enrichment analysis results.

To investigate the biological functions, a total of 1423 genes (22.23% of all DEGs) were mapped to the reference pathways in the Kyoto Encyclopedia of Genes and Genomes (Table S4). The results showed that three biological pathways were enriched (*p* < 0.05) in downregulated DEGs, including the Forkhead box protein O3 (FoxO) signaling (bmor04068), animal autophagy (bmor04140), and protein processing in endoplasmic reticulum (bmor04141) pathways. Two biological pathways were enriched (*p* < 0.05) in upregulated DEGs, including the ribosome (bmor03010) and oxidative phosphorylation (bmor00190) pathways (Figure 4, Table 2).

### 2.6. Validation of RNA Sequencing Data via Quantitative Real-Time Polymerase Chain Reaction

**Figure 5:**
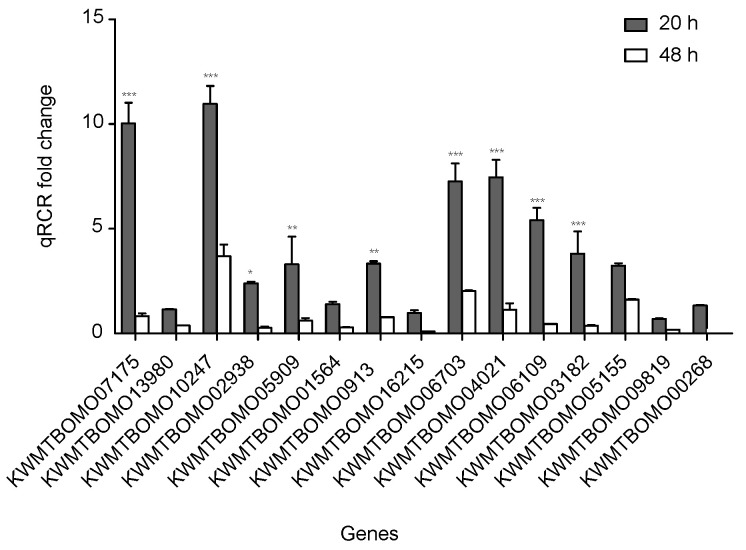
Quantitative real-time polymerase chain reaction (qPCR) of differentially expressed genes in silkworm diapause eggs at 48 h versus that at 20 h after oviposition. Relative gene expression was normalized against reference gene tif-4A (KWMTBOMO02081). *, *p* < 0.05; **, *p* < 0.01; and ***, *p* < 0.001.

To confirm the reliability of the RNA sequencing data, 15 DEGs from the FoxO signaling pathway with down-expression patterns were selected for quantitative real-time polymerase chain reaction (qPCR). All of these DEG expression patterns were consistent with the RNA sequencing data, though the fold change findings did not match exactly (Figure 5, Table S5).

## 3. Discussion

*Bombyx mori* is an economically important insect that can be effectively used to study embryonic diapause. In sericulture, eggs are usually prevented from entering diapause in favor of production demands. Thus, clarifying the mechanism of embryonic diapause is relevant to both sericultural production and scientific research. The transcriptome analysis revealed that DEGs involved in energy metabolism pathways, for example, the oxidative phosphorylation and insulin/FoxO signaling pathways, were significantly enriched, which may cause changes in energy and metabolism during diapause.

### 3.1. Oxidative Phosphorylation Pathway and ROS

As soon as diapause sets in, silkworm embryos rapidly enter a hypoxic state [15]. Hypoxia can induce increased ROS release from the mitochondrial oxidative phosphorylation chain [16]. A certain level of ROS is closely related to growth arrest in *Caenorhabditis elegans* and *Helicoverpa armigera* cotton bollworm [17,18]. In this study, ROS levels were significantly higher in diapause eggs at 48 h after oviposition than in those at 20 h. These results agree with those from our previous research, which showed that ROS levels in diapause eggs peaked within 72 h of oviposition [14]. Thus, elevated ROS levels may participate in the onset of silkworm egg diapause.

Moreover, ROS generation mainly occurs through respiratory complexes I (NADH dehydrogenase), II (succinate dehydrogenase), and III (cytochrome bc1 complex) of the oxidative phosphorylation chain, which convert O_2_ into superoxide anions (O_2_^-^) and H_2_O_2_ [16,19]. DEGs related to oxidative phosphorylation (ko00190) were mostly upregulated in diapause eggs at 48 h versus that at 20 h after oviposition: 16 of 18 complex I DEGs, 1 of 3 complex II DEGs, 3 of 4 complex III DEGs, 7 of 7 complex IV DEGs, and 23 of 24 complex V DEGs were upregulated (Table S6). GO analysis revealed that upregulated DEGs were mainly enriched in hydrogen transport (GO:0006818), proton transport (GO:0015992), ATP-related metabolism and transport (GO:0046034, GO:0006754, GO:0015986, and GO:0015988), and mitochondrial protein complex (GO:0098798). These data strongly suggested that the elevated ROS levels derived from the upregulated oxidative phosphorylation pathway, which has also been confirmed in C. elegans that increased ROS from promoting mitochondrial metabolism [20].

### 3.2. Insulin/FoxO Signaling Pathway and Carbohydrate Metabolism

**Figure 6:**
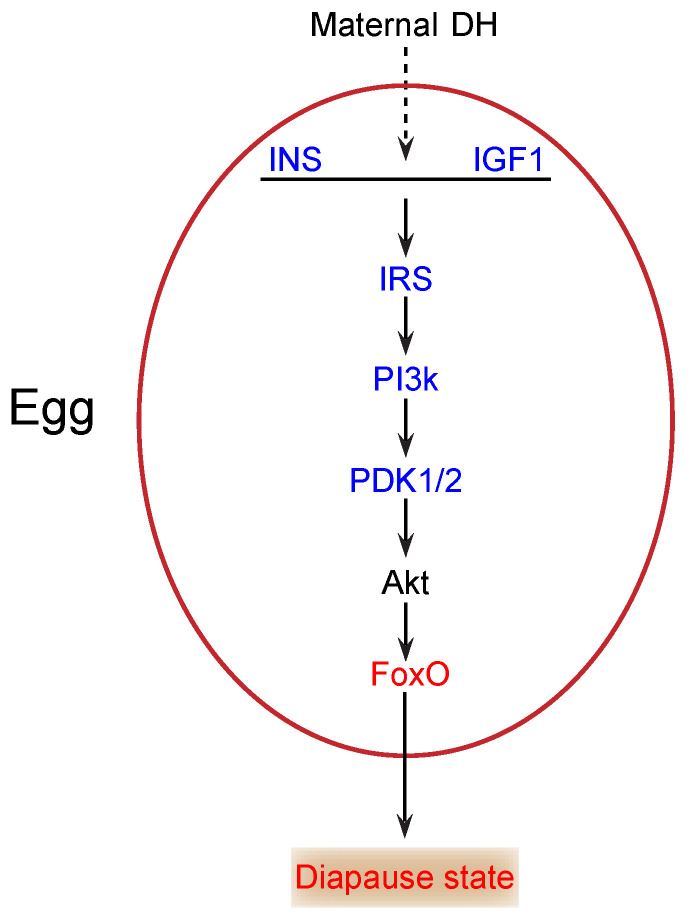
Potential insulin/FoxO signaling pathway in silkworm diapause eggs at onset of diapause. Blue text indicates downregulation, red text indicates upregulation, and black text indicates that neither up- nor downregulation was confirmed in this study. INS, insulin; IGF1, insulin-like growth factor 1; IRS, insulin receptor substrate 1-B; PI3k, phosphatidylinositol 3-kinase 60; DH, diapause hormone; PDK1/2, 3-phosphoinositide-dependent protein kinase 1; FoxO, Forkhead box protein O3.

During the onset of diapause, glycogen content changes significantly, and the total carbohydrates declines markedly [12]. Thus, carbohydrates likely play a key role as energy sources in diapause. The insulin/FoxO signaling pathway is essential in carbohydrate metabolism and growth in multicellular organisms [21,22]. In our study, insulin (INS) (KWMTBOMO00268) and insulin-like growth factor 1 (IGF1) (KWMTBOMO05156) were downregulated in diapause eggs at 48 h versus that at 20 h after oviposition (Figure 6). Previous studies demonstrate that the major function of insulin/IGF-like peptides is to regulate nutrient metabolism and stage growth [23]. During the onset of diapause, downregulation of INS and IGF1 expression suggested that the insulin signaling pathway was possibly inhibited, as eggs entering diapause required less energy. The phosphatidylinositol 3-kinase 60 (PI3k) gene, an important intermediate regulator of this pathway, is downregulated in the brains of cotton bollworms in diapause [24]. Similarly, in this study, the PI3k gene (KWMTBOMO05909 and KWMTBOMO07175) was significantly downregulated (Figure 6). The decrease in PI3k further indicated that the insulin signaling pathway was inhibited.

In addition, the 3-phosphoinositide-dependent protein kinase 1 (PDK1/2) gene (KWMTBOMO06703) was significantly downregulated in diapause eggs at 48 h versus that at 20 h after oviposition (Figure 6), which likely results in the activation of FoxO by decreasing Akt levels [25]. FoxO triggers various cellular and physiological processes, including diapause [26]. Our transcriptome data indicated that the expression of the FoxO gene (KWMTBOMO04995) was upregulated in diapause eggs at 48 h versus that at 20 h after oviposition (Figure 6), suggesting that it may induce silkworm eggs to enter diapause. Moreover, some genes in the pathway were found in GO terms related to kinase activity and phosphorus metabolic processes, such as AMP-activated protein kinase (AMPK) (KWMTBOMO09819) and cyclin-dependent kinase 2 (CDK2) (KWMTBOMO03182), which may be involved in the generation of ROS [17] and inhibition of cell cycle [27], respectively. Consequently, decreased insulin signaling and protein modification occurred in individuals at the onset of diapause [1].

### 3.3. Ribosome Pathway

Previous research has shown that the nucleoli of silkworm diapause eggs remain stable, with low ribosome synthesis during the early developmental stages after oviposition [28]. Interestingly, most DEGs of the ribosome pathway were upregulated in diapause eggs at 48 h versus that at 20 h after oviposition (Table S2). GO analysis showed that upregulated DEGs were mainly enriched in ribosomes (GO:0005840), ribonucleoprotein complexes (GO:0030529 and GO:1990904), and ribosome structural constituents (GO:0003735). It was remarkable that the core subunits of respiratory complexes are produced by mitochondrial DNA-encoded polypeptides in mitochondrial ribosomes, which consists of 79 ribosomal proteins [29]. Furthermore, DEGs related to ribonucleotide biosynthesis (GO:0009260) and ribonucleotide metabolism (GO:0009259) were upregulated, but DEGs related to the nucleus were downregulated (GO:0005634), suggesting that nucleotide metabolism may be involved in mitochondrial protein synthesis. Consequently, we speculated that the ribosomes belonged to the mitochondria and not the cytoplasm, thus meeting the production of the oxidative phosphorylation pathway. The same upregulated pathway is also found in *Locusta migratoria* diapause eggs [30].

### 3.4. Protein Processing in Endoplasmic Reticulum Pathway

Silkworm diapause embryos may depend on protein catabolism to maintain energy homeostasis. In diapause eggs, the levels of most amino acids fluctuate within 48 h after oviposition and then remain stable during diapause [31]. The newly synthesized polypeptides are transported into the endoplasmic reticulum via translocons, which consists of the Sec61 complex. Impairment of the Sec61 translocon can effectively block the production of secreted proteins in humans and *Drosophila* [32,33]. In our study, Sec61 (KWMTBOMO01918, KWMTBOMO12895, and KWMTBOMO07600) genes were significantly downregulated in silkworm diapause eggs at 48 h versus that at 20 h after oviposition. Moreover, we found that the downstream genes coding for both correctly folded and misfolded proteins were downregulated (Table S7). This caused upstream imported polypeptides to shut down. GO analysis showed that downregulated DEGs were enriched in mRNA-related metabolism (GO:0016071 and GO:0006397), protein modification (GO:0006464 and GO:0036211), and protein transport (GO:0015031, GO:0015833, and GO:0042886). Protein translation and modification were inhibited when silkworm embryos entered diapause, suggesting that their metabolism slowed to conserve energy and survive stressful conditions.

### 3.5. Autophagy Pathway

Autophagy is a self-degradation pathway that normally has a homeostatic function [34], but it may induce a cytoprotection response under various stresses. Previous studies have identified the core autophagy-related genes (ATGs): ATG8 protein homologs accumulate in starved *C. elegans*, including dauer larvae and aging adults [35]. However, in *Artemia parthenogenetica*, autophagy levels decrease drastically in diapause embryos, and the protein levels of ATG5 and ATG8 are downregulated [36]. While some researchers proposed that autophagy is upregulated to fulfil the energy requirements for survival by nutrient recycling [37], others have suggested that, under some conditions, cell cycle arrest is associated with reduced autophagy [38]. In the present study, the autophagy pathway was downregulated in silkworm diapause eggs at 48 h versus that at 20 h after oviposition. Most of the DEGs in the pathway were downregulated (Table S8), such as ATG12 (KWMTBOMO10615), ATG5 (KWMTBOMO08799), and ATG7 (KWMTBOMO12764). Our findings supported the latter notion, suggesting a response to stressful conditions during diapause.

In conclusion, we investigated DEGs during the onset of diapause and found enrichment of several signaling pathways involved in oxygen, carbohydrate, and protein metabolism. The results revealed limited available energy, slowed metabolism, and various adaptive responses at the onset of diapause in silkworm eggs, which helped the egg to prepare for lifespan extension. We also identified strong candidates for subsequent functional studies, namely, impaired insulin, elevated ROS, and activated FoxO, which may trigger diapause.

## 4. Materials and Methods

### 4.1. Sample Preparation

Bivoltine silkworms (Dazao) were provided by the State Key Laboratory of Silkworm Genome Biology. As per the approach used in our previous study [13], diapause-destined eggs were obtained through the incubation of the parental generation under long-day conditions (18L/6D h) at 25 °C during embryonic development. Samples from diapause eggs were collected at 20 and 48 h oviposition, immediately frozen in liquid nitrogen, and stored at −80 °C until use. Three samples were collected from each group.

### 4.2. RNA Extraction and RNA Sequencing

Total RNA was extracted using TRIzol reagent (Invitrogen, Waltham, MA, USA). RNA integrity was assessed using the RNA Nano 6000 Assay Kit of the Bioanalyzer 2100 system (Agilent Technologies, Santa Clara, CA, USA). The cDNA library was constructed using NEBNext^®^ Ultra™ Directional RNA Library Prep Kit for Illumina^®^ (NEB, Illumina, San Diego, CA, USA), following the manufacturer’s protocol. The library was sequenced on an Illumina Hiseq platform. The raw data were submitted to the National Center for Biotechnology Information Short Read Archive (NCBI-SRA; http://www.ncbi.nlm.nih.gov/sra/) under the accession number PRJNA327613.

### 4.3. DEGs Data Analysis

The raw data of fastq format were processed by in-house perl scripts to remove adapter sequences, reads containing ploy-N, and low quality reads. The reference genome was downloaded from the SilkBase web site (Nov.2016), using GFF annotation files (http://silkbase.ab.au-tokyo.ac.jp/cgi-bin/download.cgi). The clean reads were aligned to the reference genome using Hisat2 (v2.0.5). The mapped reads were assembled by StringTie (v1.3.3b) [39]. FeatureCounts v1.5.0-p3 was used to count the reads numbers mapped to each gene. The expression level of each gene was measured by FPKM. Differential expression analysis in diapause eggs at 48 h versus that at 20 h after oviposition (three biological replicates per condition) was performed using the DESeq2 R package (1.16.1). Genes with an adjusted *p*-value < 0.05 found by DESeq2 were assigned as differentially expressed.

### 4.4. Gene Ontology and Kyoto Encyclopedia of Genes and Genomes Enrichment Analysis

GO enrichment analysis of DEGs was implemented by interproscan software, in which gene length bias was corrected [40,41]. GO terms with corrected *p*-value less than 0.05 were considered significantly enriched by differential expressed genes. In order to explore the biological interaction among DEGs, KOBAS software was used to test the statistical enrichment of differential expression genes in KEGG pathways [42].

### 4.5. Validation of DEGs via Quantitative Real-Time Polymerase Chain Reaction

Total RNA was extracted using TRIzol (Invitrogen) and reverse transcribed to cDNA by M-MLV reverse transcriptase (Promega, Madison, WI, USA). The primers were designed using Primer Premier 5.0 (Biosoft, Palo Alto, CA, USA) and synthesized by Sangon Biotech (Shanghai, China) (Table S5). qPCR was performed using the Real-Time PCR Detection System (Bio-Rad Laboratories, Berkeley, CA, USA) with 10 µL SYBR Premix Ex Taq (TaKaRa, Osaka, Japan), 2 µL cDNA, and 0.2 µM of each primer. The thermo-cycling parameters were as follows: initial denaturation for 30 s at 95 °C and 40 cycles of 3 s at 95 °C and 30 s at 60 °C. Each sample was analyzed in triplicate. Gene expression was quantified using 2^−ΔΔCt^ method [43], with reference gene tif-4A [44] (KWMTBOMO02081) as the internal control for the normalization of data.

### 4.6. Statistical Analysis

Experimental data are shown as the mean ± standard deviation (SD) of the three replications. All data were analyzed using the GraphPad Prism 5.0 software (GraphPad Software Inc., San Diego, CA, USA). Significance was calculated using *t*-test or two-way analysis of variance (ANOVA). Differences between samples were regarded as significant at *p* < 0.05.

## Figures and Tables

**Table 1:** Statistical analysis of differentially expressed gene (DEG)seq data in silkworm diapause eggs at 20 and 48 h after oviposition.

Sample	Raw Reads	Clean Reads	Clean Base(G ^1^)	Total Map %	Q 20%	Average % GC
20h-1	57,668,622	56,668,126	8.5	97.0	98.24	42.27
20h-2	56,516,086	55,326,462	8.3	96.02	97.32	41.52
20h-3	55,698,056	54,874,326	8.23	97.07	98.27	42.77
48h-1	61,436,124	60,410,096	9.06	96.34	97.65	43
48h-2	54,909,912	53,811,608	8.07	96.15	97.94	42
48h-3	42,968,676	42,353,288	6.35	96.73	98.04	43.41

^1.^ G base = 10^9^ base.

**Table 2:** Kyoto Encyclopedia of Genes and Genomes (KEGG) pathway significant enrichment of differentially expressed genes in silkworm diapause eggs at 48 h versus that at 20 h after oviposition. FoxO, Forkhead box protein O3.

Description	KEGG ID	Term	GeneRatio ^1^	BgRatio ^2^	*p*-Value
Downregulated Pathways	bmor04068	FoxO signaling pathway	32/679	65/2777	1.04 × 10^−5^
	bmor04140	Autophagy-animal	33/679	78/2777	0.000331016
	bmor04141	Protein processing in endoplasmic reticulum	48/679	129/2777	0.000644446
Upregulated Pathways	bmor03010	Ribosome	72/744	119/2777	2.76 × 10^−15^
	bmor00190	Oxidative phosphorylation	53/744	102/2777	3.27 × 10^−8^

^1^ The ratio of the number of differential genes annotated to the KEGG pathway number to the total number of differential genes; ^2^ the ratio of the number of background genes annotated on the KEGG pathway number to the total number of background genes.

## Supplementary Materials

The following are available online at https://www.mdpi.com/1422-0067/21/17/6180/s1, Figure S1: Transcript expression level distribution in silkworm diapause eggs at 20 and 48 h after oviposition. *Y*-axis represents logarithmic values of fragments per kilobase of transcript sequence per million base pairs sequenced (FPKM), and *x*-axis represents box plot of FPKM distribution of different samples. Figure S2: Pearson correlation analysis of differentially expressed genes in silkworm diapause eggs at 48 h vs. that at 20 h after oviposition. Table S1: Fragments per kilobase of transcript sequence per million base pairs sequenced of 17,967 silkworm genes in silkworm diapause eggs at 48 h vs. that at 20 h after oviposition. Table S2: Differentially expressed genes in silkworm diapause eggs at 48 h vs. that at 20 h after oviposition. Table S3: Gene ontology (GO) term enrichment of differentially expressed genes in silkworm diapause eggs at 48 h vs. that at 20 h after oviposition. Table S4: Kyoto Encyclopedia of Genes and Genomes (KEGG) pathway enrichment of differentially expressed genes in silkworm diapause eggs at 48 h vs. that at 20 h after oviposition. Table S5: Information of the genes for quantitative polymerase chain reaction validation. Table S6: Differentially expressed genes (DEGs) of respiratory complexes in oxidative phosphorylation pathway. Table S7: Differentially expressed genes (DEGs) of correctly folded and misfolded proteins in protein processing in endoplasmic reticulum pathway. Table S8. Differentially expressed genes (DEGs) of the core autophagy-related in autophagy pathway.

## Abbreviations

Bm	Bombyx mori
DH	Diapause hormone
DEGs	Differentially expressed genes
GO	Gene ontology
ROS	Reactive oxygen species
FPKM	Fragments per kilobase of transcript sequence per million base pairs
INS	Insulin
IGF1	Insulin-like growth factor 1
PI3k	Phosphatidylinositol 3-kinase 60
PDK1/2	3-phosphoinositide-dependent protein kinase 1
AMPK	AMP-activated protein kinase
CDK2	Cyclin-dependent kinase 2
FoxO	Forkhead box protein O3
Sec61	Transport protein Sec61 alpha subunit
