# Supplementary figures and images for: Early Molecular Events during Onset of Diapause in Silkworm Eggs Revealed by Transcriptome Analysis
Source: Int J Mol Sci. 2020 Aug 27;21(17):6180. doi: 10.3390/ijms21176180 (PMC7503879; doi:10.3390/ijms21176180)

# FPKM distribution

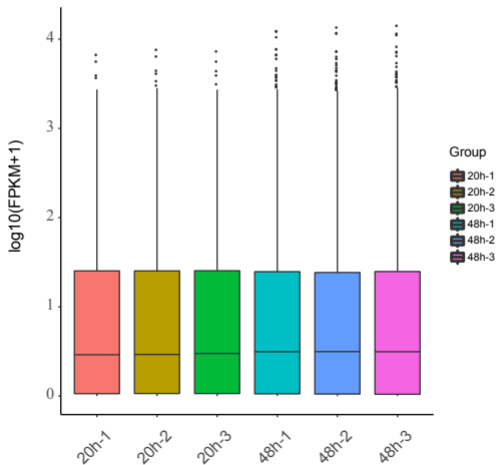

Supplement: Supplementary file 1 [file ijms-21-06180-s001.zip › supplementary files/fig.s1.pdf]

## Pearson correlation between samples

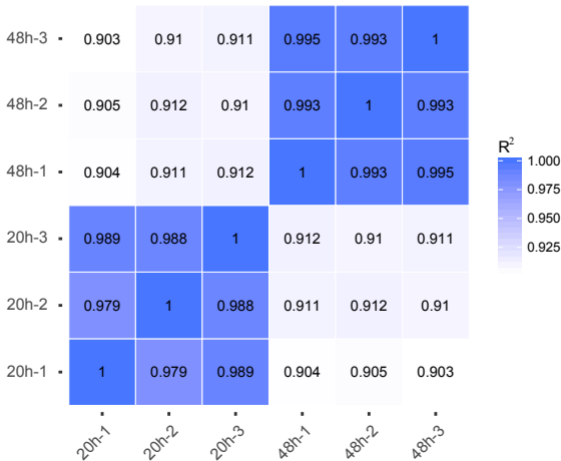

Supplement: Supplementary file 1 [file ijms-21-06180-s001.zip › supplementary files/fig.s2.pdf]
